# Autism in DSM-5: progress and challenges

**DOI:** 10.1186/2040-2392-4-13

**Published:** 2013-05-15

**Authors:** Fred R Volkmar, Brian Reichow

**Affiliations:** 1 Child Study Center, Yale University School of Medicine, PO Box 207900, New Haven, CT 06520-7900, USA

**Keywords:** Autism spectrum disorders, diagnosis, DSM-IV, DSM-5

## Abstract

**Background:**

Since Kanner’s first description of autism there have been a number of changes in approaches to diagnosis with certain key continuities . Since the Fourth edition of the Diagnostic and Statistical Manual (DSM-IV) appeared in 1994 there has been an explosion in research publications. The advent of changes in DSM-5 presents some important moves forward as well as some potential challenges.

**Methods:**

The various relevant studies are summarized.

**Results:**

If research diagnostic instruments are available, many (but not all) cases with a DSM-IV diagnosis of autism continue to have this diagnosis. The overall efficiency of this system falls if only one source of information is available and, particularly, if the criteria are used outside the research context. The impact is probably greatest among the most cognitively able cases and those with less classic autism presentations.

**Conclusions:**

Significant discontinuities in diagnostic practice raise significant problems for both research and clinical services. For DSM-5, the impact of these changes remains unclear.

## Introduction

Commonality in approaches to classification help us communicate more effectively about clinical problems (rapidly conveying a general sense of the kinds of difficulties exhibited) and conduct better research by insuring comparability of samples across sites and countries. As Rutter and Schopler noted, there is not a single, simple, right way to approach this task [1,2] and, for psychiatric and developmental disorders, a range of approaches have been developed. Official diagnostic systems, such as the World Health Organization’s International Classification of Diseases (ICD-10) and the American Psychiatric Associations Diagnostic and Statistical Manual, fourth edition (DSM-IV), have usually been oriented around specific categories but have increasingly also included dimensional approaches to provide better characterization. Before autism was first recognized officially by the Diagnostic and Statistical Manual, third edition (DSM-III) in 1980, it was very difficult to be sure of the comparability of samples; this hindered the ability to synthesize findings across studies and hampered research. Since the alignment of diagnostic criteria of DSM-IV and ICD-10, there has been an explosion of research – with well over 2,000 peer-reviewed scientific papers published last year, an increase from fewer than 2,000 peer-reviewed papers published in the *decade* before DSM-IV/ICD-10, highlighting the utility of such an alignment.

Various interests must be balanced in designing official systems; for example, reliability and ease of use, differentiation of categories, and consideration of developmental issues [3]. Probably the major difference between ICD-10 and DSM-IV is the provision in ICD-10 of separate guidelines for research and clinical work while DSM-IV provides one set of guidelines for both purposes. Other differences include issues of comorbidity, impairment requirements, and implications for service eligibility.

This paper reviews recent developments in the diagnosis of autism in Diagnostic and Statistical Manual, fifth edition (DSM-5). We begin with a short review of the history of diagnostic approaches, the rational for the DSM-5 model, and a discussion of its uses and limitations.

### Diagnosis of autism from Kanner to DSM-IV

The condition known as autistic disorder, childhood autism, or infantile autism was first described by Kanner in his report of 11 children with what appeared to him to be a novel condition characterized by two essential features of autism; a lack of interest in the social world, and a group of behaviors he referred to as ‘resistance to change’ or ‘insistence on sameness’ [4]. Kanner’s thoughtful clinical description noted many of the features still commonly included in diagnostic criteria for the disorder, and his emphasis on the centrality of social difficulties remains a hallmark of the condition. Early research was confused by some false leads and a lack of clarity about the validity of autism (as compared with childhood schizophrenia). By the 1970s longitudinal and other studies strongly suggested the validity of the condition, its frequent association with intellectual disability, and its strong brain [5] and genetic basis [6].

As research accumulated it also became clear that language-communication problems were a major source of disability, so that by the late 1970s there was a consensus that autism was characterized by: impaired social development of a type quite different from that in normal children; impaired language and communication skills – again of a distinctive type; resistance to change or insistence on sameness, as reflected in inflexible adherence to routines, motor mannerisms, and stereotypies, and other behavioral oddities; and an onset in the first years of life. Available research strongly supported inclusion of autism as a new condition in DSM-III, which had adopted an atheoretical research diagnostic criteria approach [3].

In DSM-III autism was included in a class of conditions called pervasive developmental disorder (PDD); this term had the advantage of no previous history. The DSM-III definition was very focused on infantile autism and developmental change was only cursorily addressed, although other categories for late-onset autism were also included (although without much justification) [3].

In DSM-III-R (1987) there was a major attempt to address the lack of developmental orientation. A single disorder and a subthreshold category were described (the name of the latter was changed from atypical PDD to pervasive developmental disorder not otherwise specified (PDD-NOS)). The criteria set was more extensive than in DSM-III and included a polythetic definition with symptoms chosen from social, communication, and resistance to change categories. Although a field trial was conducted it was limited in some ways and, in retrospect, DSM-III-R appeared to overdiagnose autism in individuals with greater cognitive disability while, to some extent, underdiagnosing at the other end of the IQ range [3].

For DSM-IV a number of preliminary steps were undertaken, including invited reviews of the literature, data reanalysis, and, finally, a large multi-site field trial conducted with over 100 raters of nearly 1,000 cases in various (20+) sites around the world. Goals for DSM-IV included balancing sensitivity and specificity across the IQ range and age, convergence (if possible) with the ICD-10 diagnostic approach, and potentially including new disorders in the DSM-IV definition [7].

The final definition for autism was polythetic, had a good balance of sensitivity and specificity, and improved reliability in less experienced evaluators. DSM-IV also recognized three disorders new to DSM: childhood disintegrative disorder, Asperger’s disorder, and Rett’s disorder along with the usual subthreshold PDD-NOS category. Of these conditions, the definition of Asperger’s proved most problematic (the text was radically changes at the time DSM-IV-TR appeared but the criteria could not be changed at that point). As a result the concept has been used inconsistently although research on it has increased dramatically [8]. Rett’s disorder was included because it appeared to be a very interesting condition that might have a specific neurobiological basis and the PDDs seemed the best place for it [9]; subsequently, a gene has been discovered for this condition and it is often no longer regarded as an autism spectrum disorder, although draft versions of DSM-5 have included it as a specifier. Childhood disintegrative disorder was of great interest, despite its rarity, in that a child would develop typically to 4 years, 5 years, or even 6 years of age before having a rapid and dramatic regression followed by a more classic autism presentation [2]. DSM-IV/ICD-10 did come to convergent definitions, and the approach has been widely used and highly productive for research. This approach also facilitated the development of new dimensional approaches for screening and diagnosis that further enhanced research.

## Review

### DSM-5 and autism

As with DSM-IV, the task of revision for DSM-5 was taken seriously by the overall work group and members of the committee on neurodevelopmental disorders. Several key executive decisions adopted for all of DSM-5 have had serious implications; these are the decision to eliminate subthreshold categories and the high reliance on diagnostic instruments as a source of criteria and as proof of validity [10]. Issues more specific to autism include the nature of the revision and decisions about how best to outline and organize the proposed criteria and evaluate them.

Some aspects of the DSM-5 approach to autism appear to be well reasoned and quite valuable; for example, the move to a better description of the class of disorder (autism spectrum disorder to replace PDD) and the use of dimensions in combination with categorical approaches. Some of the more practical problems, however, probably arise in the context of the revision process, and the final product has been the subject of much debate [11,12].

Early on, the decision was made to drop the multiple disorders included in DSM-IV in favor of a single autism spectrum term. A second disorder, social (pragmatic) communication disorder, was added, although its relation to the autism spectrum disorders (ASD) remains unclear (ASD must be ruled out in the diagnostic criteria, but prevalence estimates of ASD have included social (pragmatic) communication disorder). Also unclear is the application and use of this diagnosis in practice and the types of services to which an individual might be entitled. In combination with the use of better specifiers of dimension it was hoped that clinical needs would be well served. Given its single-gene etiology, a decision was also made to remove Rett’s disorder from DSM, although an individual with this genetic condition meeting diagnostic criteria for ASD would still receive an ASD diagnosis probably with a specifier. This is a complicated precedent given the many strong leads it has provided in uncovering the genetics of autism. While a case clearly could have been made for refining the Asperger’s label, the work group elected to eliminate it as a category along with childhood disintegrative disorder. In some respects both moves are controversial, particularly given the inconsistency with which the Asperger’s diagnosis has been utilized (itself a problem but potentially one obscuring a potentially important clinical distinction). Based on a factor analysis of a large body of data from diagnostic instruments a decision was made to collapse social and communication features into a single category and then to have a second category more consistent with Kanner’s ‘insistence on sameness’/restricted interests package with the addition of a sensory sensitivity symptom, which had poor specificity in the DSM-IV field trial [7,13].

Factor analysis methods have their uses and limitations – depending at the most basic level on which data are included in the analysis and how the analysis is constrained. For the DSM-IV field trial either a two-factor, three-factor or five-factor solution could be derived; others performing similar analyses have noted the complexity of these approaches [14]. The final decision for DSM-IV and ICD-10 to keep the traditional three categories (dating back to Rutter’s 1978 definition [15]) was made partly for reasons of historical continuity and, strategically, having three categories of criteria gave many different combinations of criteria that would yield an autism diagnosis (well over 2,000).

There is no question that social and communicative features are very closely related, but the problem in combining them into one category results in many fewer potential criteria combinations. Another factor contributing to the decreased combinations of symptoms is the move back to a monothetic approach for the social communication domain, where instead of two out of four criteria and one out of four criteria required in the DSM-IV, three out of three criteria are required in the DSM-5. A polythetic approach was retained for the repetitive and restrictive behavioral domain, although the number of symptoms needing to be met was increased from one out of four (zero of four potentially in PDD-NOS) to two out of four. We are not advocating for one approach over the other; they each have advantages and disadvantages and are often used together for diagnostic criteria [16]. Rather, we are illustrating the impact that this decision is likely to have on the composition of the autism spectrum, which might more closely resemble the more classic autism described by Kanner [4] than the broader autism spectrum that might be captured with polythetic criteria. An additional consequence of requiring all three social criteria could be the delayed diagnosis (and consequentially the delay of intervening) for children whose symptoms do not fully manifest until social demands increase.

Data from a large series of well-characterized cases were used to produce the draft DSM-5 criteria for the single new autism spectrum disorder. Two research diagnostic instruments (one a parent report measure and the other observational assessment) were used [17]. The authors rightly noted that this was not a field trial, and their results suggested that when both the parent interview and child assessment were conducted, sensitivity/specificity were maximized; however, in the absence of both, specificity fell. Their data suggested that no more than about 10% of cases would lose their diagnosis. Other data on reliability were also provided from a field trial focused only on this issue [18], although the overall approach to the DSM-5 field trial has also been criticized [19,20]. Other data using large datasets have also provided some support to the approach undertaken [21]. Given the data available and the major effort undertaken, then, what are the potential problems?

### Issues in use of DSM-5

Despite the name change to autism spectrum disorder, the concept actually proposed is apparently more restricted than the DSM-IV approach. A series of papers using different approaches and different samples suggest that the issue may be more extensive than would otherwise be thought. If the results of these studies are realized, there would probably be large implications for service eligibility and research for individuals currently receiving support for the disorder. It is important to note in moving DSM-5 into what may be more real-world clinical settings that practitioners will not likely have had the extensive training in diagnostic instruments.

The results of most of the relevant independent studies can be succinctly summarized. It is important to note that in some cases studies were conducted using an earlier version of DSM-5 and that diverse methods and samples were used. Mattila and colleagues used a slightly earlier draft of DSM-5 to assess agreement with DSM-IV [22]. In this epidemiological study a very large sample of 8-year-old children were assessed using the Autism Spectrum Screening Questionnaire and 110 were then seen for more in-depth assessment. The investigators noted that DSM-5 was less sensitive than DSM-IV. Comparisons were also made between DSM-IV and DSM-5, showing individuals with higher IQs being less likely to meet the new diagnostic criteria.

Similarly, Worley and Matson compared symptoms of ASD in several hundred children using DSM-IV and DSM-5. Significant differences were noted in terms of core domain scores on socialization/communication between DSM-IV and DSM-5 [23]. In both cases the groups had significantly higher levels of dysfunction than a control group and the number no longer meeting criteria in DSM-5 was noted to be a potential problem for both clinical service provision and research; for example, relative to epidemiological or longitudinal studies.

Frazier and colleagues evaluated proposed DSM-5 criteria in a large sample of siblings (some with ASD and others without ASD) [21]. They noted that in this sample of children (ages 2 to 18) the specificity of DSM-5 was higher than that of DSM-IV while sensitivity was lower and that relaxing the diagnostic threshold might improve the approach.

McPartland and colleagues reanalyzed a large sample of cases selected from the DSM-IV field trial [24]. Sensitivity and specificity were systematically assessed using a symptom checklist approach to cross-walk DSM-IV criteria to DSM-5. The specificity of DSM-5 was high (94.9%) but sensitivity varied dramatically by clinical group (varying from 0.76 in autism to 0.25 in Asperger’s disorder and 0.28 in PDD-NOS) and by cognitive ability (IQ <70 = 0.70; IQ ≥70 = 0.46).

Mattson and colleagues examined alternative approaches to improve DSM-5 [25]. They evaluated two potential modifications for toddlers, with some degrees of overall improvement but with significant numbers of toddlers apparently left without eligibility for services. They noted that while excluded from the diagnostic categories these toddlers continued to exhibit significant impairment.

Gibbs and colleagues compared DSM-IV-TR and DSM-5 diagnosis in a sample of 132 children [26]. Of the 111 who had received a diagnosis of autism or related PDD in DSM-IV-TR, 26 did not meet the criteria in DSM-5; most of those excluded from ASD in DSM-5 would have received a diagnosis of PDD-NOS in DSM-IV-TR.

Taheri and Perry reviewed over 130 cases of children with previous diagnosis of autism or PDD-NOS and found that about 60% met the new DSM-5 criteria (81% of those with autism but less than 20% of those with PDD-NOS) [27]. They also noted a significant relationship to IQ, with more able cases more frequently losing a diagnosis.

Most recently, Wilson compared DSM-IV, ICD-10, and DSM-5 in a sample of 150 adults with ASD who were more cognitively able [28]. The author noted that about 56% of those meeting ICD-10 also met DSM-5 (although nearly 20% of those not meeting criteria for ASD met DSM-5 criteria for social communication disorder). They noted that this might be an important practical problem in terms of access to service and suggested modification either in the diagnostic threshold (reducing the number of criteria required) or in giving greater leniency to uncertain criteria (allowing them to count). While the first of these proposals would be relatively easy to implement, the second poses significant difficulty. As the authors emphasized, exclusion from clinical services is a potentially significant problem.

On the other hand, Mazefsky and colleagues compared DSM-IV and DSM-5 diagnosis using the research instruments on which the new DSM-5 approaches is based [29]. They found that 93% of the nearly 500 high-functioning participants met the criteria for autism in DSM-5 but that this number was lower when only the parent instrument used and lower still if only the individual assessment was available. While reassuring from the point of view of research, if both instruments are available it is worrisome that, in practice, for adults a parental informant may not be available.

## What do all these results mean?

As noted previously there are some very sensible and praiseworthy aspects of DSM-5. The decision to eliminate subcategories is controversial, and the important thing is that individuals who need and benefit from services are still able to obtain these services. It is also imperative that we do not have major changes in research diagnosis. This would pose a significant challenge for many studies; for example, those with epidemiological or longitudinal samples, or studies of treatments that span decades. It is also unclear what changes will be made in ICD-11 and a lack of alignment of the international and American definitions could impact research. It would seem to be important to base what may be significant change on a very solid body of data.

One might ask what we really know the actual impact of DSM-5 will be. The simple response is that we do not know [30]. As we have outlined, a series of studies suggest that many children might no longer meet the diagnostic criteria of ASD. Less is known about very young children, which we would like to capture early to provide early intervention services when the brain has its most plasticity, or adults, who are an understudied population in which little is known regarding best practice. A very recent study has suggested that the DSM-5 approach is overly restrictive with this age group as well and became adequate only when scoring rules were modified [31].

## Conclusions

Since the appearance of DSM-IV in 1994, research on autism and related conditions has expanded dramatically. Part of this expansion reflects the consensus on diagnosis exemplified by the convergence of DSM-IV and ICD-10 and a more flexible diagnostic approach than employed by its predecessors. As with any human construction there is no question that sensible changes can and should be made. On the other hand, there is also a tension around being overly enthralled with change given the potential for complicating past work, ongoing longitudinal and epidemiological studies, and service provision.

While some of the changes employed in the new DSM-5 approach are praiseworthy, others are much more complicated. There appears to be some significant potential for diagnostic change, essentially as – despite what might conceptually appear to be a broader tent of the autism spectrum – the DSM-5 approach seems likely to result in a narrower concept. This raises some concern about the impact on services for children in need as well as for comparison with previous research. Sadly we are, to a considerable extent, still in the dark on the extent of this change. Although the focus on standardized diagnostic instruments has some important advantages in the real world of clinics and schools, clinicians will not have had the opportunity to undertake extensive training. In some cases the new (but relatively unclear) social communication disorder concept may be invoked, but the lack of research on this putative condition poses other problems and its use might well be taken as an excuse to avoid service provision.

These are clear areas where future research will be needed. Aside from these unknowns, it seems likely that some proportion of the cases will lose their label and thus potentially lose their eligibility for services. If this proves correct, then this is a drastic change both from the service and research points of view. While some aspects of the new approach clearly have considerable merit, the lack of data on this most fundamental issue is problematic. It is possible that the issue is much less significant for research centers where trained examiners are available and when both parent interview and individual assessment research instruments are available. However, given that the Diagnostic and Statistical Manual is intended for use in the actual clinical world (for example, a psychiatrist’s practice in a suburban American town, a pediatric nurse conducting a level-one screener in an inner-city health clinic on a 18-month-old child, at a 10-minute 4-year annual evaluation), the issue of its impact on eligibility remains to be seen. We will only know this after prospective studies conducted in community-based sites (not university clinics) report comparisons between DSM-IV and DSM-5 criteria. Hopefully once DSM-5 has appeared and is used in practice, these issues will be clarified and, if need be, revisions can rapidly be made.

## Abbreviations

ASD: autism spectrum disorders; DSM-5: Diagnostic and Statistical Manual, 5th edition; DSM-III: Diagnostic and Statistical Manual, 3rd edition; DSM-III-R: Diagnostic and Statistical Manual, 3rd edition revised, DSM-IV, Diagnostic and Statistical Manual, 4th edition; DSM-IV-TR: Diagnostic and Statistical Manual, 4th edition text revision, ICD-10, International Classification of Diseases, 10th edition; PDD: pervasive developmental disorder; PDD-NOS: pervasive developmental disorder not otherwise specified.

## Competing interests

The authors declare that they have no competing interests.

## Authors’ contributions

FRV and BR contributed to the first draft of the manuscript, proofed the manuscript, and read and approved the manuscript.
